# Changing perspectives: attempting to de-colonize the gaze of a Canadian medical student

**Published:** 2016-12-05

**Authors:** Elaine Bradley

**Affiliations:** 1 Faculty of Medicine, University or Toronto

Sitting in a circle of women, participating in a traditional Indigenous* ceremony†, I feel my hands begin to sweat. The tobacco in my left palm darkens in color as it sticks to the growing beads. It has come to the time in the ceremony when all women present introduce themselves. I listen as one by one, they speak about their origins, the tribe and clan of their family, and how they came to be here in this room, together. My anxiety builds as my turn gets closer and closer. Myself, a 4th year medical student with no Indigenous heritage, I am faced with the difficult questions of who am I? And how did I come to be *here?*

In my final year of medical school, I undertook an elective focused on urban Indigenous health. I had a certain impression and idea of the learning I was going to gain, as the health inequities faced by Indigenous peoples in Canada are extensively documented.^2^,^17^–^19^ I was both confused and surprised when my first task as part of my elective was to locate Indigenous community events and find some to attend. I remember walking into a drum social, awkwardly finding a seat, and anxious about how the evening would unfold. As I watched people gather to eat, dance, and socialize, I was left feeling unsure of what my objective was; what knowledge was I to gain from this encounter?

Much of medical school involves observation: watching a lecture or procedure, sitting in on a patient interview. Our place as an observer is formalized within the medical system, with “observer-ships” and “shadowing” acting as both mandatory curricular components and elective experiences. The intent is to observe a physician, and, in turn, observe patients, utilizing the living model of illness in order to learn how to identify and diagnose. Rarely, if ever, is the impact of our observation on these objects, these people, discussed.

Gaze theory, originating in both psychoanalytic and feminist theory, postulates the idea of “gazing” as a “one way event that denies the agency of the perceived object.”^11^ In Visual Pleasure and Narrative Cinema (1989), Laura Mulvey describes the concept of the “male gaze” in film;she describes how the male, as both the creator and observer of the film, uses his position of power and privilege to oppress and objectify the female subjects on screen.^14^ Foucault (1994) describes the historical emergence of the “medical gaze;” describing how patients in Paris in the 18th Century were transformed into objects to be studied as they entered the physician’s office.^5^ Foucault suggested that the “medical gaze” established a power relationship between the physician (the “gazer”) and the patient(the object of this “gaze”).^5^ Foucault was not the first to reference this power imbalance, David T. Goldberg (1993) observes that “the neutrality and objectifying distantiation of the rational scientist” creates “the theoretical space for a view to develop subjectless bodies”.^8^ He expands upon this to say, that once a person is “objectified” they could be “analyzed, categorized, classified, and ordered with the cold gaze of scientific distance.”^8^

The “study” of Indigenous people in Canada by the medical system has a long and difficult history, including the extensive experimentation that occurred as part of the residential school system, as “bureaucrats, doctors, and scientists... increasingly came to view Aboriginal bodies as “experimental materials.”^14^ In more recent years, epidemiological studies of Indigenous communities have examined the inequities in health for Indigenous Canadians, but have “depicted Aboriginal and Native American peoples as sick, powerless, and lacking in capacity, information that is used to reinforce unequal power relations, paternalism, and dominance and to undermine their aspirations for sovereignty” resulting in communities expressing concern and resistance to outsider research.^12^

As this (certainly not exhaustive) set of examples illustrates, Foucault’s concept of a “medical gaze” is too simple to be applied in Canada; it fails to incorporate the intersectionality of the objects it looks upon. Gilman (1985) writes that “medical icons... are iconographic in that they represent these realities in a manner determined by the historical position of the observers, their relationship to their own time, and to the history of the conventions which they deploy”.^7^ This idea is expanded upon by Yancy (2008), who argues that colonized peoples are subject to the “colonial gaze”, a historical perspective stemming from European colonizers who were able to “discern with “clarity” and “accuracy” the “truth” about certain human bodies vis-à-vis a white racist discursive regime of truth.”^16^ He continues on to suggest that “this racist form of colonial establishment precludes mutual recognition as equals.”

The existence of a “colonial gaze” in the medical literature is less clearly defined. The concept of a formalized “medical student gaze” is addressed by Davenport (2000) in her analysis of medical student involvement in a U.S. clinic for the homeless. She suggests that when students were outside formal medical institutions they were able to manifest “social reflexivity—scrutiny and revision of social beliefs and practices.”^4^ Davenport’s analysis represents a positive outlook, indicating that students, outside the formalized objectivity of educational institutions are able to break down their “medical gaze.” Yet, not unlike Foucault, her analysis falls short in its simplicity, limiting the discussion of identity to that of socioeconomic status, and failing to incorporate the impact of institutionalized colonialism on the “medical gaze.” As such, her lack of reflexivity in this parameter limits the applicability of this analysis. In contrast, Bleakley *et al.* (2008) identify a colonial gaze in the process of the globalization of Westernized medical education, stating that educators in the metropolitan West working internationally “risk continuing the process of colonisation despite [their] good intentions.”^3^ In the nursing literature, Racine (2011) in her examination of international nursing placements, echoes similar themes addressing the “potential for educational and scientific exploitation.”^15^ Despite the authors’ emphasis on reflexivity and examining gaze, the articles both focus entirely on international interactions and do not look at medical education as an agent of colonisation within Western nations themselves. Still, a common theme throughout these articles suggests a process of awareness and self-reflexivity among health care practitioners, but how can this translate into something that can be taught to medical students?

Self-location, an Indigenous research methodology, involves the “protocol of locating oneself within Indigenous communities and research,” suggesting that through “honest self-location and admitting what one does not know, researchers build trust with research participants.”^6^ Gillies*et al.* suggest that the process of self-location can be uncomfortable, requiring the researcher to engage in “decolonization processes that include honesty about who we are and from where we come.”^6^ For me, self-location means stating I am a third generation Canadian, Caucasian(of Scottish and English descent), cisgender female, born in Alberta and residing in Toronto. I am also a medical student, studying at a formal academic institution, through which I am attempting to study “Indigenous health.”

Personally, the process of self-location, and in turn, the analysis of the components of my “gaze” left me unsure of how to feel. At my first drum social, far away from the hospital or clinic, I became acutely aware of my position as someone “gazing” at others, attempting to objectively gain knowledge by looking. With my increased understanding of the complex relationship between indigenous Canadians and the healthcare system, I became very self-conscious that as a medical student, simply by being present in a space, I could make it feel less safe for others. This self-location challenged me to understand medicine as a one of the possible “many agencies of white power and knowledge...that function as vehicles through which white hegemony is further expressed and maintained” a concept that had never been addressed in my classes or placements.^16^

When it finally came to my turn in the traditional ceremony†, I felt nervous, but also better prepared to self-locate (to articulate my understanding of where I am coming from, and my awareness of the sociocultural impact my presence brings into a space). Armed with an increased knowledge of power relationships and indigenous methodologies, I therefore expressed my gratitude at how I was welcomed into the circle and admitted my lack of knowledge of how to participate in a respectful way. his learning experience represented a much-needed shift in my medical education. Here, I rejected the concept of learning solely through objectivity and exposed myself to learning experiences outside of institutionalized power structures. I was privileged to participate in the components of this elective that exposed me to another culture, but am wary of recommending it as a mandatory component of a curriculum, as I must remember that we cannot put the onus of those subjected to the “gaze” to correct it for us. In addition, it is important to acknowledge that the self-location process for medical students is unique to every individual and their own background. I believe that the process of self-location is not a static endeavour, it is an ongoing effort to understand and acknowledge the complexity and impact of my engagement in the provision of healthcare in Canada. So how do I and others, as members of the medical institution, work to create a safer environment for Indigenous Canadians? To start, in her essay Situated Knowledges, Donna Harraway argues that in order to reject the concept of “scientific objectivity,” the “object of knowledge be pictured as an actor and agent, not as a screen or a ground or a resource.”^10^ From my experience, I believe that being given the resources and space to self-locate within the medical system and the greater societal context is the first step for medical students to begin to break down the many factors that contribute to “medical observation” as an agent of colonialism. As medical education becomes increasingly globalized, with many Canadian students engaging in placements abroad, and international students studying here in Canada, the importance of self-location as a component of the medical curriculum cannot be understated. Yet this form of self-reflexivity cannot occur solely on an individual student level. The learning and space for medical students to examine and deconstruct their perspective, I believe, is an essential part of Canadian medical education, which perhaps can only be achieved when academic medical institutions perform the groundwork to locate themselves.

## Footnotes

* In this paper the word “Indigenous” is used as an inclusive term to describe those in Canada who self-identify as or have relation to “First Peoples,” including but not limited to First Nations peoples, Métis and Inuit.

† The names, origins, and specifics of any traditional Indigenous ceremonies referenced in this essay have been purposefully omitted to protect both the privacy of those traditions and the individuals present.
